# The Impacts of Genistein and Daidzein on Estrogen Conjugations in Human Breast Cancer Cells: A Targeted Metabolomics Approach

**DOI:** 10.3389/fphar.2017.00699

**Published:** 2017-10-05

**Authors:** Stefan Poschner, Alexandra Maier-Salamon, Martin Zehl, Judith Wackerlig, Daniel Dobusch, Bettina Pachmann, Konstantin L. Sterlini, Walter Jäger

**Affiliations:** ^1^Division of Clinical Pharmacy and Diagnostics, Department of Pharmaceutical Chemistry, University of Vienna, Vienna, Austria; ^2^Department of Analytical Chemistry, Faculty of Chemistry, University of Vienna, Vienna, Austria; ^3^Division of Drug Design and Medicinal Chemistry, Department of Pharmaceutical Chemistry, University of Vienna, Vienna, Austria; ^4^Vienna Metabolomics Center (VIME), University of Vienna, Vienna, Austria

**Keywords:** soy, genistein, daidzein, breast cancer, estrogens, metabolomics

## Abstract

The beneficial effect of dietary soy food intake, especially for women diagnosed with breast cancer, is controversial, as *in vitro* data has shown that the soy isoflavones genistein and daidzein may even stimulate the proliferation of estrogen-receptor alpha positive (ERα+) breast cancer cells at low concentrations. As genistein and daidzein are known to inhibit key enzymes in the steroid metabolism pathway, and thus may influence levels of active estrogens, we investigated the impacts of genistein and daidzein on the formation of estrogen metabolites, namely 17β-estradiol (E2), 17β-estradiol-3-(β-D-glucuronide) (E2-G), 17β-estradiol-3-sulfate (E2-S) and estrone-3-sulfate (E1-S) in estrogen-dependent ERα+ MCF-7 cells. We found that both isoflavones were potent inhibitors of E1 and E2 sulfation (85–95% inhibition at 10 μM), but impeded E2 glucuronidation to a lesser extent (55–60% inhibition at 10 μM). The stronger inhibition of E1 and E2 sulfation compared with E2 glucuronidation was more evident for genistein, as indicated by significantly lower inhibition constants for genistein [K_i_s: E2-S (0.32 μM) < E1-S (0.76 μM) < E2-G (6.01 μM)] when compared with those for daidzein [K_i_s: E2-S (0.48 μM) < E1-S (1.64 μM) < E2-G (7.31 μM)]. Concomitant with the suppression of E1 and E2 conjugation, we observed a minor but statistically significant increase in E2 concentration of approximately 20%. As the content of genistein and daidzein in soy food is relatively low, an increased risk of breast cancer development and progression in women may only be observed following consumption of high-dose isoflavone supplements. Further long-term human studies monitoring free estrogens and their conjugates are therefore highly warranted to evaluate the potential side effects of high-dose genistein and daidzein, especially in patients diagnosed with ERα+ breast cancer.

## Introduction

Breast cancer is the most prevalent cancer in women and the second leading cause of cancer-related deaths among females worldwide (Ferlay et al., 2015). Chemoprevention in combination with anticancer treatment is therefore crucial to reduce rates of morbidity and mortality. Evidence from epidemiological and experimental studies indicates that several natural products may act as chemopreventive agents and inhibit mammary carcinogenesis (Pan et al., 2015). Among these products is soy, which contains variable amounts of genistein and daidzein as the major isoflavones (approximately 47 and 44%, respectively) and minor amounts of glycitein (approximately 9% of the total isoflavones in soybeans). The genistein and daidzein content is therefore also predominant in soy-derived foods and dietary supplements (Wiseman et al., 2002; Clarke et al., 2008).

Epidemiological studies have indicated that soy intake post-diagnosis not only improves prognosis but is also associated with statistically significant reductions in breast cancer recurrence (Kang et al., 2010; Chi et al., 2013). However, based on the weak estrogen-like effects of the isoflavones genistein and daidzein, some researchers and clinicians are concerned that a high soy intake may increase the cancer risk. Indeed, *in vitro* studies have shown that both genistein and daidzein stimulate the proliferation of MCF-7 human estrogen-receptor alpha positive (ERα+) breast cancer cells at low concentrations, but inhibit tumor growth at higher doses. In ER alpha negative (ERα-) cells (MDA-MB-231), this biphasic effect is not observed; both phytoestrogens exhibit an anti-proliferative effect only. This indicates that the proliferative effect of genistein and daidzein, as observed at low doses, is ERα-mediated, while ERβ, which is expressed at low levels in both MCF-7 and MDA-MB-231 cells, seems to oppose ERα actions and exhibits anti-migratory and anti-invasive properties (Vladusic et al., 2000; Al-Bader et al., 2011; Wang et al., 2012; Uifãlean et al., 2016).

Besides an ERα-mediated interaction, low concentrations of both isoflavones can also induce cell proliferation via G protein coupled estrogen receptor 1 (GPER1) by stimulating cAMP production, intracellular Ca^2+^ mobilization and cSrc activation. Subsequently, the transactivation of the epidermal growth factor receptor (EGFR) is triggered, leading to an activation of downstream signaling pathways such as PI3K/Akt and MAPK/ERK (Uifãlean et al., 2016).

In addition to ERα interaction and activation of signaling pathways, the stimulatory effect of genistein and daidzein on ERα+ breast cancer cells might also be linked to increased steroid hormone levels, which drive cellular proliferation and thus are an important factor for carcinogenesis (Folkerd and Dowsett, 2013). Mechanism for altered steroid levels between ERα+ and ERα- breast cancer cells include differences in estrogen metabolism. Indeed, incubation of human ERα+ MCF-7 breast cancer cells with E1 for 24 h resulted in a more than sevenfold higher formation of estrogen sulfates compared to ERα- MDA-MB-231 cells, as cellular SULT expression is significantly higher in MCF-7 cells (Pasqualini, 2009). However, estrogen conjugates do not promote ER-mediated activity but represent a local reservoir of native E1 and E2 after hydrolysis by sulfatases (Samavat and Kurzer, 2015). Interestingly, a previous human trial demonstrated that participants who consumed a high-soy diet for 13 months showed a non-significant increase of urinary E2 levels of 18% (Maskarinec et al., 2012), suggesting a possible role of E2 in the observed increased cellular growth of ERα+ breast tumors by genistein and daidzein.

This prompted us to hypothesize that genistein and daidzein might dose-dependently alter steroid hormone levels by inhibiting the conjugation of estrogens and their precursors. Our hypothesis was supported by *in vitro* studies showing that soy isoflavonoids are able to inhibit various enzymes involved in the metabolism of estrogens, including cytochrome P450 3A4 (CYP3A4), 17β-HSD, SULTs, and UGTs (Mesía-Vela and Kauffman, 2003; Mohamed and Frye, 2011; Ronis, 2016; Cassetta et al., 2017).

Whether soy components influences the estrogen metabolism is not yet known. Therefore, the aim of the present study was to investigate the impacts of genistein and daidzein on estrogen metabolism in human ERα+ MCF-7 breast cancer cells. For this purpose, a newly established specific and sensitive analytical LC-HRMS assay was conducted to simultaneously quantify the main steroids of the estrogenic pathway namely E1, E2, estriol (16α-OH-17β-estradiol, E3), E1-S, E2-G and E2-S (Rižner, 2013; Mueller et al., 2015). The outcomes of metabolism were subsequently correlated with cell growth in order to better understand the effects of soy isoflavones in ERα+ breast cancer.

## Materials and Methods

### Materials

16α-hydroxy-17β-estradiol (E3), E2, 17β-estradiol-3-(β-D-glucuronide) sodium salt (E2-G) and E1, as well as acetic acid, acetonitrile, ammonium acetate, DMSO, genistein and daidzein, were purchased from Sigma–Aldrich Chemical Co. (Munich, Germany). 17β-estradiol-3-sulfate sodium salt (E2-S) and estrone-3-sulfate sodium salt (E1-S) were obtained from Steraloids, Inc. (Newport, RI, United States). 16α-hydroxy-17β-estradiol-2,4,17-d_3_ (E3-d_3_), 17β-estradiol-2,4,16,16-d_4_ (E2-d_4_), 17β-estradiol-16,16,17-d_3_-3-(β-D-glucuronide) sodium salt (E2-G-d_3_), 17β-estradiol-2,4,16,16-d_4_-3-sulfate sodium salt (E2-S-d_4_), estrone-2,4,16,16-d_4_ (E1-d_4_) and estrone-2,4,16,16-d_4_-3-sulfate sodium salt (E1-S-d_4_) were purchased from C/D/N Isotopes, Inc. (Pointe-Claire, QC, Canada). Purified water was obtained using an arium^®^pro ultrapure water system (Sartorius AG, Göttingen, Germany).

### Cell Proliferation Studies

MCF-7 breast cancer cells were purchased from the American Type Culture Collection (ATCC; Rockville, MD, United States). All experiments were performed during the exponential growth phase of the cell line. MCF-7 cells were routinely cultivated at 37°C (95% humidity and 5% CO_2_) in phenol red-free Dulbecco’s Modified Eagle Medium F-12 (DMEM/F-12; Invitrogen, Karlsruhe, Germany), fortified with 1% PenStrep^®^-solution and 10% fetal bovine serum (Invitrogen). For experimental conditions, cells were seeded at a density of 1.0 × 10^6^ cells per well and allowed to attach for 24 h. Prior to the application of genistein and daidzein, cells were washed twice with DPBS (Invitrogen), and DMEM/F-12, containing 10% HyClone^®^heat-inactivated charcoal-stripped fetal bovine serum (THP Medical Products, Vienna, Austria), was subsequently added to exclude external hormones. To evaluate the potential influence of genistein and daidzein on MCF-7 cell proliferation, cells were incubated for 48 h with E1 (100 nM) as an estrogen precursor in the presence of 1, 5, and 10 μM genistein or daidzein, respectively. Genistein, daidzein and E1 were dissolved in DMSO prior to their addition to the cell medium to give a final DMSO concentration of 0.05%. Prior to cell counting with a Coulter^®^ Z1 cell counter (Beckman Coulter GmbH, Krefeld, Germany), supernatant medium was removed and cells were detached using 400 μl TrypLe^®^ solution (Invitrogen). The effect of E1 (10, 25, 50, 75, and 100 nM) on the growth of MCF-7 cells was also determined using the same protocol as for genistein and daidzein. All experiments were performed in triplicate and the data are reported as means ± SD of all values.

### Inhibition Studies

MCF-7 breast cancer cells were cultivated in the presence of HyClone^®^ heat-inactivated charcoal-stripped fetal bovine serum as described above, and then treated with increasing concentrations of E1 (10, 25, 50, 75, and 100 nM) in the presence of 1, 5, and 10 μM genistein or daidzein, respectively. After 24 and 48 h, 2000 μl media aliquots were mixed with 20 μl deuterated internal standard solution and pre-cleaned by SPE on Oasis HLB 1 cc SPE cartridges (30 mg; Waters Corporation, Milford, MA, United States), as described previously (Poschner et al., 2017). Briefly, reconditioning of the cartridges was achieved using 2 × 1.0 ml acetonitrile and 3 × 1.0 ml ammonium acetate buffer (10 mM, pH = 5.0). Subsequently, samples were loaded onto the SPE cartridges and washed with 1 × 1.0 ml ammonium acetate buffer (10 mM, pH = 5.0) and 2 × 1.0 ml acetonitrile/ammonium acetate buffer (10 mM, pH 5.0) 10:90 (v/v). Analytes were then eluted using 2 × 650 μl acetonitrile/ammonium acetate buffer (10 mM, pH = 5.0) 95:5 (v/v), evaporated to dryness, and reconstituted in 270 μl acetonitrile/ammonium acetate buffer (10 mM, pH = 5.0) 25:75 (v/v). After media collection, cells were washed five times with 2.0 ml DPBS, detached using 200 μl TrypLE^®^ solution (37°C, 15 min), mixed with 800 μl DPBS and transferred into plastic vials. Aliquots of these suspensions (100 μl each) were used to determine the exact number of cells per sample well. For this, the aliquots were diluted 100-fold and counted using a Coulter^®^ Z1 cell counter. To additionally quantify cytosolic steroid levels, the remaining cell suspensions (900 μl each) were gently centrifuged (1000 rpm, 8 min) and the supernatants were discarded. The cell pellets were subsequently resuspended in 100 μl aqueous ammonium acetate buffer (10 mM, pH = 5.0) and lysed by five freeze-thaw-cycles in liquid nitrogen (3 min each), followed by thawing at ambient temperature. Ammonium acetate buffer (1000 μl) was then added and the suspensions were centrifuged (14000 rpm, 5 min), and the clear supernatants were concentrated using the SPE protocol described above. All processed samples were then stored at -80°C until further LC-HRMS analysis. Four biologically independent experiments were performed, and reported values represent the overall means ± SD of all determinations.

### LC-HRMS Assay

E1, E1-S, E2, E2-S, E2-G, and E3 were quantified using an LC-HRMS assay, validated according to the ICH Q2(R1) guidelines as described previously (Poschner et al., 2017). LC was performed using an UltiMate 3000 RSLC-series system (Dionex; Thermo Fisher Scientific, Inc., Germering, Germany) coupled to a maXis HD ESI-Qq-TOF mass spectrometer (Bruker Corporation, Bremen, Germany). Solvent A was aqueous ammonium acetate buffer (10 mM, pH = 5.0) and solvent B was acetonitrile. A Phenomenex Luna^®^ 3 μm C18(2) 100 Å LC column (250 mm × 4.6 mm I.D.; Phenomenex, Inc., Torrance, CA, United States), preceded by a Hypersil^®^ BDS-C18 guard column (5 μm, 10 mm × 4.6 mm I.D.; Thermo Fisher Scientific, Inc.) and maintained constantly at 43°C, was used for steroid separation at a flow rate of 1 ml/min. The injection volume was 100 μl for each sample. The gradient used was as follows: 25% solvent B at 0 min, 56.3% solvent B at 19 min, a washing step at 90% solvent B from 19.5 to 24.0 min and column re-equilibration with 25% solvent B from 24.5 to 30.5 min. The ESI ion source settings were as follows: Capillary voltage, -4.5 kV; nebulizer, 1.0 bar N_2_; dry gas flow rate, 8.0 l/min N_2_; and dry temperature, 200°C. The ion transfer parameters were set to 400 Vpp funnel RF and 300 Vpp multipole RF, the quadrupole ion energy was 8.0 eV and the collision cell parameters were as follows: Collision energy, 10.0 eV; collision RF, 1100 Vpp; transfer time, 38 μs; and pre-pulse storage, 18 μs. Full-scan mass spectra were recorded in the range of *m/z* 150–500. Control samples consisting of unspiked cell culture medium showed no detectable background traces of the analyzed hormones. To ensure accurate quantification results, quality control samples, containing each analyte at a concentration of 6-fold or 600-fold of the respective lower limits of quantification (LLOQs), were analyzed in triplicate with each LC batch. The LLOQs were determined as follows: E1, 19.0 pg/ml; E1-S, 4.0 pg/ml; E2, 140.9 pg/ml; E2-S, 3.4 pg/ml; E2-G, 12.0 pg/ml; E3, 28.4 pg/ml cell medium.

### Data Analysis

Liquid chromatography-high resolution mass spectrometry data were analyzed using Compass DataAnalysis 4.2 and QuantAnalysis 2.2 software (Bruker Corporation). EICs were created for each analyte and internal standard pair, from which the respective peak areas were determined to calculate the analyte/internal standard ratios for quantification. Kinetic analysis of estrogen metabolite formation in the presence and absence of genistein or daidzein using E1 as an estrogen precursor (10–100 nM for 48 h) was performed. All data best fitted to the Michaelis–Menten model: *V* = *V*_max_ × [*S*] / (*K*_m_ + [*S*]), where *V* is the rate of the reaction, *V*_max_ is the maximum reaction velocity, *K*_m_ is the Michaelis constant and [*S*] is the substrate concentration. Kinetic parameters were calculated using GraphPad Prism 6.0 software (GraphPad Software Inc., La Jolla, CA, United States). Inhibition modes were determined from Lineweaver–Burk plots, and corresponding *K*_i_ were calculated by plotting the slopes of the primary Lineweaver–Burk plots against the respective inhibitor concentrations using GraphPad Prism 6.0. The same software package was used for statistical analyses. All values were expressed as means ± SD and the Student’s *t*-test and ANOVA with Tukey’s post-test were used to compare differences between control samples and treatment groups. The statistical significance level was set to *P* < 0.05.

## Results

### Influence of Estrone and Soy Isoflavones on MCF-7 Cell Proliferation

**FIGURE 1:**
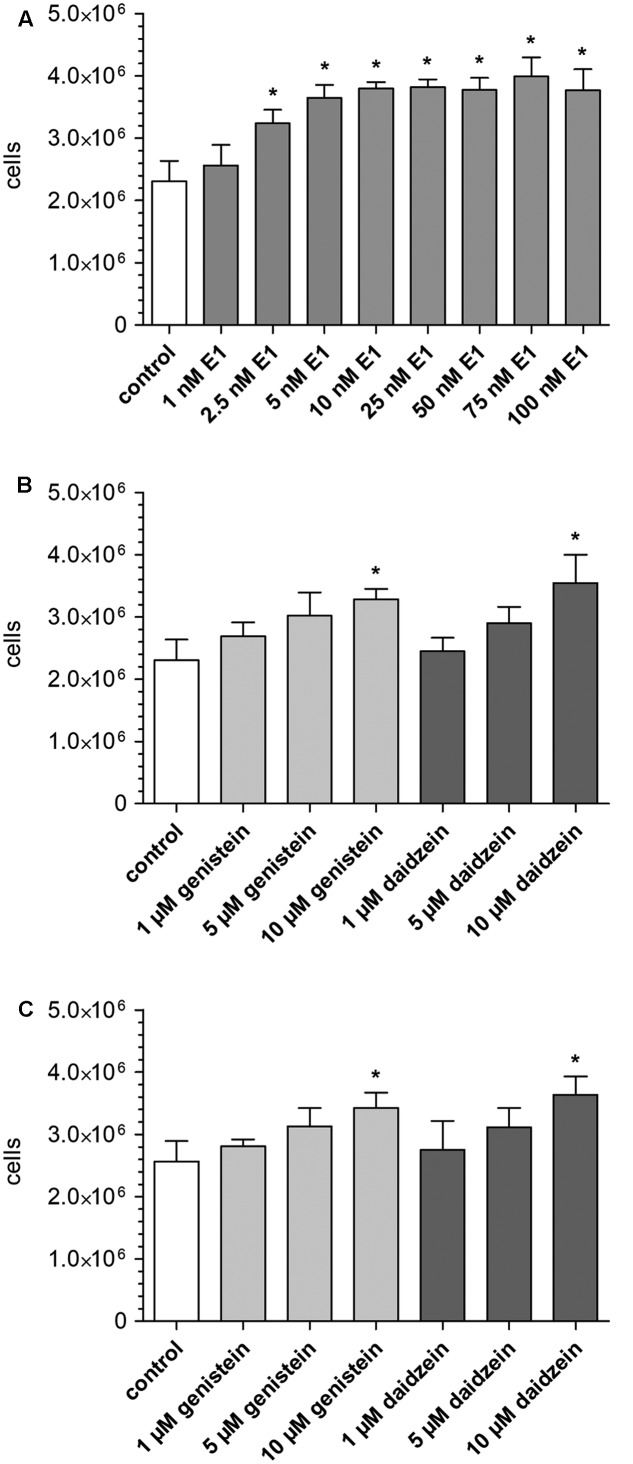
Influence of E1 and soy isoflavones on MCF-7 breast cancer cell proliferation. Cells were incubated for 48 h with **(A)** increasing concentrations of E1 (1–100 nM; control: DMSO), **(B)** increasing concentrations of genistein and daidzein (1–10 μM; control: DMSO) and **(C)** increasing concentrations of genistein and daidzein (1–10 μM) in the presence of 1 nM E1 (control: 1 nM E1 in DMSO). All data represent the means ± SD of three independent biological replicates. Asterisks (^∗^) indicate significantly different mean values in comparison to the controls (*P* < 0.05).

To assess the influence of E1 on MCF-7 cancer cell growth, cells were incubated with increasing concentrations of E1 for 48 h, detached by application of TrypLe^®^ solution and counted using a Coulter^®^ Z1 cell counter. Compared with control samples (containing DMSO only), the number of viable MCF-7 cells was significantly increased in the presence of E1 (2.31 ± 0.33 × 10^6^ vs. 3.99 ± 0.31 × 10^6^ cells) (**Figure 1A**), confirming the hormone-dependency of the MCF-7 cell line. The observed proliferative effect of E1 on the breast cancer cells was concentration-dependent with a mean maximum cell growth increase of 73% at 75 nM E1. Additionally, we evaluated the effect of E1 in combination with the soy isoflavones genistein or daidzein on MCF-7 cell growth. For this purpose, cells were co-incubated with 0, 1, 2.5, and 100 nM E1 as a hormone precursor in the presence of increasing concentrations of genistein or daidzein (1–10 μM). These concentrations were chosen as they represent plasma levels measured after the administration of isoflavone supplements. As shown in **Figure 1B**, the presence of both isoflavones in the absence of E1 had a significant effect on cell proliferation (+42% for genistein and +54% for daidzein). Co-incubation with the isoflavones and 1 nM E1 led to similar results with a mean cell number increase of +34% for genistein and +42% for daidzein (**Figure 1C**). When the E1 concentration was further increased to 2.5 nM, we observed only a slightly higher cell growth (+16 and +15%, respectively) compared to the incubation with E1 alone. In the presence of 100 nM E1, genistein and daidzein did not show any further increase of cellular growth because of the already high cell number stimulated by E1.

### Formation of Estrogen Metabolites by MCF-7 Cells

**FIGURE 2:**
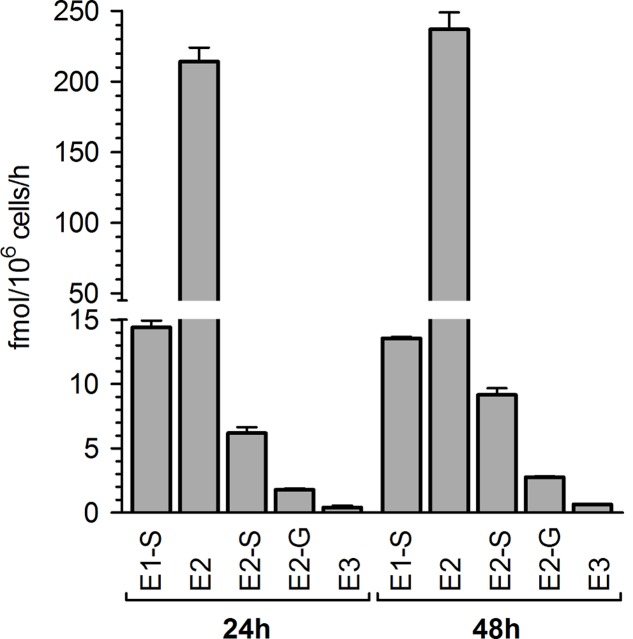
Pattern of estrogen metabolite formation rates by MCF-7 cells. Cells were incubated with 100 nM E1 as a hormone precursor, and medium aliquots were analyzed for estrogen metabolites at 24 and 48 h. Data represent the means ± SD of three independent biological replicates.

In preliminary experiments, MCF-7 breast cancer cells were treated with 100 nM E1 as a hormone precursor and cell media aliquots were analyzed for E1 and its metabolites after 24 and 48 h. By using a highly specific and sensitive LC-HRMS assay, five biotransformation products could be quantified besides the precursor E1 in the cellular medium (**Figure 2**). As metabolite formation showed a linear trend with time up to 48 h, incubations in all further experiments were finalized after this time-span as it ensured the most precise quantification of the biotransformation products.

E2 represented the main metabolite in the cellular supernatant, with a mean formation rate of 233.1 ± 6.9 fmol/10^6^ cells/h after 48 h (**Figure 2** and **Table 1**). It was further sulfated and glucuronidated to E2-S and E2-G, with a markedly favoritism for the sulfated product (9.15 ± 1.21 vs. 2.76 ± 0.37 fmol/10^6^ cells/h). In addition to the conjugation reactions, E2 was also hydroxylated to E3, though this was a minor metabolite in MCF-7 cells with a formation rate of only 0.65 ± 0.05 fmol/10^6^ cells/h. Alongside the two E2 conjugates, we also observed the sulfation of the precursor E1 to E1-S (13.5 ± 2.1 fmol/10^6^ cells/h). Concomitant with the formation of these five metabolites, the E1 concentration in the medium decreased by 29% from 100 nM to 71.0 ± 0.9 nM after 48 h. The total molar proportion of all five metabolites was 28.9% indicating that these five biotransformation products represent almost 100% of all metabolites formed from the precursor E1 by the MCF-7 cells (un-metabolized E1 + total detected metabolites: 99.9%). Interestingly, intracellular metabolite concentrations in all samples were below the respective detection limits (data not shown).

**Table 1:** Estrogen metabolism by MCF-7 cells in the presence of genistein and daidzein.

Inhibitor	E2 (fmol/10^6^ cells/h)	E1-S (fmol/10^6^ cells/h)	E2-S (fmol/10^6^ cells/h)	E2-G (fmol/10^6^ cells/h)
Control	233.1 ± 6.9	13.5 ± 2.1	9.15 ± 1.21	2.76 ± 0.37
1 μM genistein	242.1 ± 16.7	10.2 ± 1.8	**5.90 ± 0.76^∗^**	**2.12 ± 0.34^∗^**
5 μM genistein	**264.0 ± 8.8^∗^**	**2.26 ± 0.27^∗^**	**1.12 ± 0.37^∗^**	**1.41 ± 0.33^∗^**
10 μM genistein	**277.2 ± 18.2^∗^**	**1.27 ± 0.22^∗^**	**0.54 ± 0.05^∗^**	**1.06 ± 0.10^∗^**
1 μM daidzein	244.2 ± 9.0	**8.15 ± 1.26^∗^**	**5.91 ± 1.27^∗^**	2.39 ± 0.27
5 μM daidzein	**260.7 ± 19.2^∗^**	**3.11 ± 0.34^∗^**	**1.27 ± 0.34^∗^**	**1.94 ± 0.30^∗^**
10 μM daidzein	**275.4 ± 5.9^∗^**	**2.00 ± 0.30^∗^**	**0.69 ± 0.16^∗^**	**1.26 ± 0.15^∗^**


*Cells were incubated with increasing concentrations of the soy isoflavones genistein and daidzein and 100 nM E1 as hormone precursor for 48 h. All data represent the means ± SD of four independent biological replicates. Values in bold and marked with an asterisk (^∗^) are significantly different in comparison to the control values (*P* < 0.05).*

Kinetic profiles for the formation of estrogen metabolites by MCF-7 cells were then evaluated over an E1 concentration range of 10–100 nM for 48 h. 17β-HSD-mediated formation of the main metabolite E2 best fitted to the Michaelis–Menten model, with a mean *V*_max_ value of 464.5 ± 39.2 fmol/10^6^ cells/h and a mean *K*_m_ value of 95.4 ± 14.0 nM (**Table 2**). E2-S and E2-G formation also exhibited Michaelis–Menten kinetics with similar *K*_m_ values (95.9 ± 5.4 and 92.7 ± 9.0 nM, respectively), though the *V*_max_ value for sulfation was 3.3-fold higher than that for glucuronidation (18.3 ± 0.7 vs. 5.52 ± 0.37 fmol/10^6^ cells/h), confirming the preference for E2 sulfation by MCF-7 cells. Kinetic parameters calculated for the sulfation of the precursor E1 (*V*_max_ = 26.8 ± 2.3 fmol/10^6^ cells/h, *K*_m_ = 88.3 ± 11.3 nM) were comparable to those for E2 sulfation, probably because both are substrates of the same enzyme isoforms SULT1A1 and SULT1E1 (Harris et al., 2004). Evaluation of the kinetic profile for E3 formation was not possible, as only the highest E1 concentration (100 nM) but not the lower ones, resulted in E3 concentrations above the LLOQ of the presented assay.

**Table 2:** Kinetic parameters of estrogen metabolism by MCF-7 cells in the presence of genistein and daidzein.

Inhibitor	E2		E1-S		E2-S		E2-G	
				
	*V*_max_ (fmol/10^6^ cells/h)	*K*_m_ (nM)	*V*_max_ (fmol/10^6^ cells/h)	*K*_m_ (nM)	*V*_max_ (fmol/10^6^ cells/h)	*K*_m_ (nM)	*V*_max_ (fmol/10^6^ cells/h)	*K*_m_ (nM)
Control	464.5 ± 39.2	95.4 ± 14.0	26.8 ± 2.3	88.3 ± 11.3	18.3 ± 0.7	95.9 ± 5.4	5.52 ± 0.37	92.7 ± 9.0
1 μM genistein	463.3 ± 39.7	87.1 ± 13.4	**19.1 ± 2.0^∗^**	84.1 ± 12.9	**13.5 ± 0.6^∗^**	98.2 ± 4.5	**4.13 ± 0.34^∗^**	91.3 ± 10.9
5 μM genistein	520.7 ± 32.9	95.2 ± 10.5	**4.28 ± 0.42^∗^**	91.6 ± 12.9	**2.19 ± 0.07^∗^**	96.8 ± 4.5	**2.84 ± 0.20^∗^**	106.4 ± 10.3
10 μM genistein	**543.9 ± 31.6^∗^**	93.8 ± 9.51	**2.58 ± 0.25^∗^**	93.9 ± 13.3	**1.04 ± 0.07^∗^**	97.8 ± 9.8	**2.16 ± 0.06^∗^**	106.3 ± 9.1
1 μM daidzein	488.2 ± 36.4	96.4 ± 12.4	**17.1 ± 1.4^∗^**	102.6 ± 11.5	**11.9 ± 0.9^∗^**	101.2 ± 10.8	**4.83 ± 0.27^∗^**	98.0 ± 7.8
5 μM daidzein	508.7 ± 22.7	95.6 ± 7.39	**6.43 ± 0.28^∗^**	104.4 ± 14.3	**2.64 ± 0.22^∗^**	102.8 ± 11.9	**3.99 ± 0.18^∗^**	100.4 ± 6.6
10 μM daidzein	**543.5 ± 32.9^∗^**	96.8 ± 10.1	**4.20 ± 0.47^∗^**	105.7 ± 16.5	**1.35 ± 0.11^∗^**	98.6 ± 11.9	**2.63 ± 0.32^∗^**	105.6 ± 18.3


*Cells were incubated with increasing concentrations of the soy isoflavones genistein and daidzein and 10 to 100 nM E1 as hormone precursor for 48 h. All data represent the means ± SD of four independent biological replicates. Values in bold and marked with an asterisk (^∗^) are significantly different in comparison to the control values (*P* < 0.05).*

### Inhibition of Estrogen Conjugations by Genistein

To assess the possible inhibitory effect of genistein on estrogen metabolism, MCF-7 cells were first treated with E1 (100 nM) for 48 h in the presence and absence of increasing genistein concentrations (1, 5, and 10 μM). As shown in **Table 1**, a marked inhibition of E1 and E2 conjugation by this isoflavone was observed (Supplementary Figure S1A). Even in the presence of 1 μM genistein, the formation rates of E1-S, E2-S and E2-G decreased by approximately 25–35% compared to control. At 10 μM genistein, the inhibition was more obvious, and more pronounced for sulfation than for glucuronidation. The formation rates of E1-S and E2-S were reduced by approximately 90–95%, respectively, compared with the control (E1-S, 1.27 ± 0.22 vs. 13.5 ± 2.1 fmol/10^6^ cells/h; E2-S, 0.54 ± 0.05 vs. 9.15 ± 1.21 fmol/10^6^ cells/h), whereas E2-G formation was only reduced by approximately 60% (1.06 ± 0.10 vs. 2.76 ± 0.37 fmol/10^6^ cells/h). Genistein also showed a pronounced inhibition on E3 formation (data not shown); based on its low concentration in the cellular medium, the corresponding *K*_i_ could not be calculated.

**FIGURE 3:**
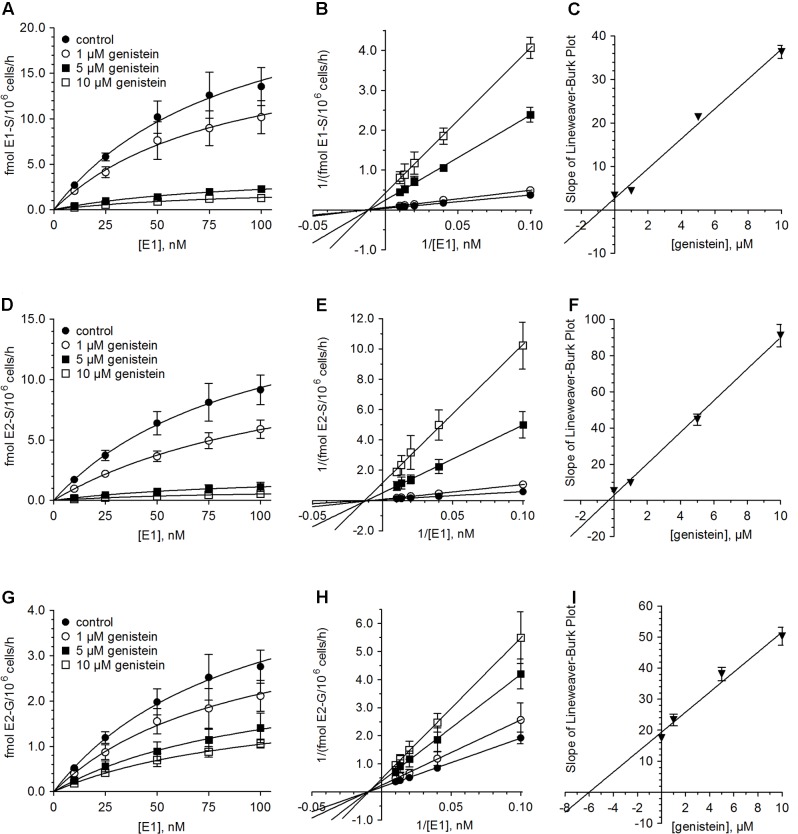
Inhibition of estrogen conjugation by genistein. The kinetics of **(A–C)** E1 sulfation, **(D–F)** E2 sulfation and **(G–I)** E2 glucuronidation were calculated following incubation of MCF-7 cells with 10 to 100 nM E1 for 48 h in the presence (1–10 μM) and absence of genistein. Data is displayed in Michaelis–Menten, Lineweaver–Burk and *K*_i_ value plots. All data represent the means ± SD of four independent biological replicates.

In order to determine the kinetic parameters for the observed inhibition processes, cells were treated with 10–100 nM E1 as hormone precursor and increasing concentrations of genistein (1–10 μM). As shown in **Figure 3**, the presence of genistein did not alter the kinetic profiles for E1 or E2 conjugations; the data still best-fitted to the Michaelis–Menten kinetic model. However, the mean *V*_max_ values for the formation of the conjugates were significantly decreased by increasing genistein concentrations, while the corresponding *K*_m_ values were almost unaffected (**Table 2**). In order to gain further insight into the inhibition process, we determined the mode of inhibition by genistein on E1 and E2 metabolism and calculated the corresponding *K*_i_ by plotting the slopes of the primary Lineweaver–Burk plots against the respective inhibitor concentrations (**Figure 3**). As reported in **Table 3**, non-competitive inhibition by genistein was confirmed for all E1 and E2 conjugates, as initially indicated by the altered *V*_max_ values and the almost unchanged *K*_m_ values (**Table 2**). The more pronounced inhibition of E1 and E2 sulfation over E2 glucuronidation by genistein (**Figure 3**) was also reflected by significantly lower *K*_i_ values for sulfation (E2-S, 0.32 μM and E1-S, 0.76 μM vs. E2-G, 6.01 μM), indicating decreased rates of sulfate formation even at very low genistein concentrations.

**Table 3:** Inhibition constants (*K*_i_) and modes of inhibition.

Isoflavone	Metabolic activity	*K*_i_ (μM)	Mode of inhibition
Genistein	E1 sulfation	0.76	Non-competitive
	E2 sulfation	0.32	Non-competitive
	E2 glucuronidation	6.01	Non-competitive
Daidzein	E1 sulfation	1.64	Non-competitive
	E2 sulfation	0.48	Non-competitive
	E2 glucuronidation	7.31	Non-competitive


**K*_i_ values were obtained by plotting the slopes of the respective Lineweaver–Burk plots against isoflavone concentration. All data represent the means ± SD of four independent biological replicates.*

### Inhibition of Estrogen Conjugations by Daidzein

Analogous to the cell experiments conducted with genistein, we first investigated the possible inhibition of E1 and E2 metabolism by daidzein. Again, the formation rates of E1-S, E2-S and E2-G were significantly decreased by approximately 15–40% compared to control, even at 1 μM daidzein (**Table 1** and Supplementary Figure S1B). The increase in daidzein concentration to 10 μM led to more pronounced suppression of E1 and E2 sulfation by approximately 85 and 90% compared to control (E1-S, 2.00 ± 0.30 vs. 13.5 ± 2.1 fmol/10^6^ cells/h; E2-S, 0.69 ± 0.16 vs. 9.15 ± 1.21 fmol/10^6^ cells/h), while E2 glucuronidation was only reduced by approximately 55% (1.26 ± 0.15 vs. 2.76 ± 0.37 fmol/10^6^ cells/h). Like genistein, also daidzein showed a pronounced inhibition of the very minor metabolite E3 (data not shown). However, based on its low concentration in the cellular medium, we were not able to calculate the kinetics for its inhibition.

**FIGURE 4:**
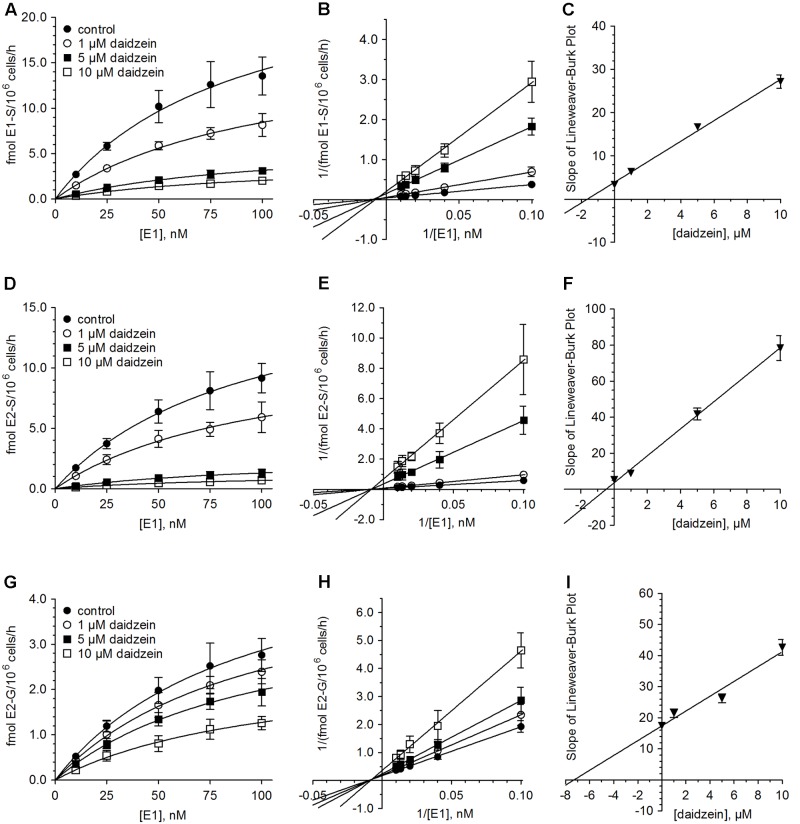
Inhibition of estrogen conjugation by daidzein. The kinetics of **(A–C)** E1 sulfation, **(D–F)** E2 sulfation and **(G–I)** E2 glucuronidation were calculated following incubation of MCF-7 cells with 10 to 100 nM E1 for 48 h in the presence (1–10 μM) and absence of daidzein. Data is displayed in Michaelis–Menten, Lineweaver–Burk and *K*_i_ value plots. All data represent the means ± SD of four independent biological replicates.

**Figure 4** and **Table 2** show the Michaelis–Menten parameters for the formation of E1-S, E2-S, and E2-G by MCF-7 cells in the presence of increasing daidzein concentrations (1–10 μM). The *V*_max_ and *K*_m_ values were comparable to those calculated for the inhibition by genistein. The observed decrease in *V*_max_ values and unaltered *K*_m_ values, together with the corresponding Lineweaver–Burk plots, again indicated that a non-competitive mechanism was the most likely mode of inhibition by daidzein. As shown in **Table 3**, the *K*_i_ values were also significantly lower for sulfation compared with glucuronidation [E2-S (0.48 μM) < E1-S (1.64 μM) < E2-G (7.31 μM)].

### Effect of Genistein and Daidzein on E2 Formation

**FIGURE 5:**
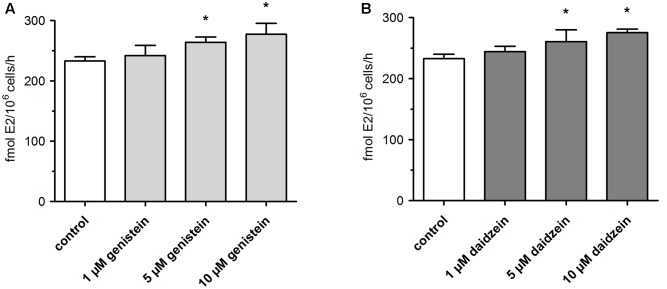
Formation of active E2 by MCF-7 breast cancer cells. Cells were incubated with 100 nM E1 in the presence of **(A)** genistein or **(B)** daidzein. All data represent the means ± SD of four independent biological replicates. Asterisks (^∗^) indicate significantly different mean values in comparison to the controls (*P* < 0.05).

Concomitant with the observed suppression of SULT- and UGT-mediated conjugation of E1 and E2, we observed a minor but statistically significant increase in E2 formation (**Figure 5**). When MCF-7 cells were exposed to 100 nM E1 in the presence of 1 μM isoflavone, E2 levels were elevated by approximately 4–5% (242.1 ± 16.7 fmol/10^6^ cells/h for genistein and 244.2 ± 9.0 fmol/10^6^ cells/h for daidzein) compared to control (233.1 ± 6.9 fmol/10^6^ cells/h). Inhibition of E1 and E2 conjugation with 10 μM genistein or daidzein further increased E2 formation by ∼20% compared to control (277.2 ± 18.2 fmol/10^6^ cells/h for genistein and 275.4 ± 5.9 fmol/10^6^ cells/h for daidzein) (**Table 1**).

In parallel, *V*_max_ values increased from 464.5 ± 39.2 fmol/10^6^ cells/h to 543.9 ± 31.6 or 543.5 ± 32.9 fmol/10^6^ cells/h, when cells were treated with 10 μM genistein or daidzein, respectively. As already shown for E1 and E2 conjugates, *K*_m_ values were not affected by either isoflavone.

## Discussion

To the best of our knowledge, the present study was the first to investigate the concentration-dependent impacts of the soy isoflavones genistein and daidzein on the formation of estrogen conjugates in human ERα+ breast cancer cells (MCF-7). When cells were exposed solely to the hormone precursor E1, proliferation increased up to 1.6-fold. A stimulatory effect on cell proliferation was also observed when cells were incubated with genistein or daidzein (1–10 μM) in the absence or in the presence of low E1 concentrations (1 and 2.5 nM). Our findings are in line with previous studies that have also reported a stimulatory effect of these isoflavone concentrations on MCF-7 cell growth (Chen et al., 2015; Wei et al., 2015). Our data also correlate with the *in vitro* study by Kuiper et al. (1998), which shows that genistein presents a 20- to 30-fold higher binding affinity for ERβ than for ERα while daidzein has only a fivefold increased affinity for ERβ, explaining the observed slightly increased proliferative effect of daidzein on ERα+ MCF-7 cell growth (**Figure 1**).

After an incubation-period of 48 h, we were able to observe the formation of five metabolites, namely E1-S, E2, E2-S, E2-G and E3. Based on Michaelis–Menten parameters, the predominant metabolite was E2 (*V*_max_, 464.5 ± 39.2 fmol/10^6^ cells/h), while the conjugates E1-S, E2-S and E2-G exhibited significantly lower *V*_max_ values. Estimations of *K*_m_ values for E1-S, E2, E2-S and E2-G gave comparable results, indicating similar affinities to 17β-HSD, SULTs and UGTs. The CYP3A4-mediated hydroxylation of E2 to E3, however, represented only a very minor metabolic pathway by MCF-7 cells, as the formation of E3 could not be quantified at E1 concentrations of <100 nM.

Sulfation is therefore the main conjugation pathway of estrogens in MCF-7 cells as it accounted for 8.64% of total metabolites rate compared to only 1.05% for glucuronidation. The CYP3A4-mediated formation of E3 is negligible with a proportion of only 0.25% of the total E1 metabolism. These data are in line with previous *in vitro* investigations, which revealed a more than sevenfold higher formation of estrogen sulfates in human ERα+ MCF-7 breast cancer cells than ERα- MDA-MB-231 cells after incubation with E1 for 24 h, based on significantly higher SULT expression (Pasqualini, 2009). Higher SULT expression in ERα+ breast tumors compared to ERα- breast cancer tissues was also found in human primary tumor tissue samples (Adams et al., 1979).

When the cells were incubated with E1 in the presence of soy isoflavones (up to 10 μM), estrogen conjugations were markedly decreased. Genistein inhibited E1-S and E2-S formation by 90 and 95% compared to control, while E2 glucuronidation was less affected and only decreased by 60%. Interestingly, daidzein, which differs from genistein only by the absence of a hydroxyl group in position 5, showed slightly weaker inhibitory effects (E1-S, 85%; E2-S, 90%; E2-G, 55%). These findings are in accordance with previous data, which also reported a stronger inhibition of E1 and E2 sulfation by genistein than by daidzein, potentially due to a higher potency of this isoflavone against SULT1A1 (Mesía-Vela and Kauffman, 2003). Kinetic analysis in combination with corresponding Lineweaver–Burk plots showed that both isoflavones non-competitively inhibited estrogen conjugations by MCF-7 cells, with very low *K*_i_ values for E1 sulfation (genistein, 0.76 μM; daidzein, 1.64 μM). E2 sulfation was affected to an even greater extent by either isoflavone (genistein, 0.32 μM; daidzein, 0.48 μM). By contrast, the calculated *K*_i_ values for E2 glucuronidation were markedly higher (genistein, 6.01 μM; daidzein, 7.31 μM), confirming the stronger impact of isoflavone treatment on sulfation compared with glucuronidation. Non-competitive inhibition of E1 and E2 metabolite formation by both isoflavones is of clinical importance, as it suggests that the extent of inhibition depends only on the inhibitor concentration (indicated by marked decreases in *V*_max_) and not on the binding of E1 and E2 to the respective enzymes (indicated by largely unaltered *K*_m_ values).

Whether dietary soy intake or high-dose isoflavone supplements may cause or exacerbate breast cancer in post-menopausal women remains controversial. Although soy food and its isoflavones have been widely investigated in the past few decades as cancer chemopreventives, conflicting data regarding their efficacy and safety have been reported. Population-based studies (Nechuta et al., 2012; Zhang et al., 2017) have indicated beneficial effects of dietary soy food consumption for women diagnosed with ERα- breast cancer, such as reduced risk of mortality and improved treatment outcomes; however, these effects have not been observed in patients expressing ERα. Clinical trials have also raised concerns that isoflavone intake may drive cancer cell proliferation (Shike et al., 2014) and significantly increase the Ki-67 labeling index in premenopausal women (Khan et al., 2012). Therefore, understanding of the metabolic interplay between genistein, daidzein and the concentration of active E2, which is associated with breast cancer risk and progression (Folkerd and Dowsett, 2013), is crucial for risk assessments.

**FIGURE 6:**
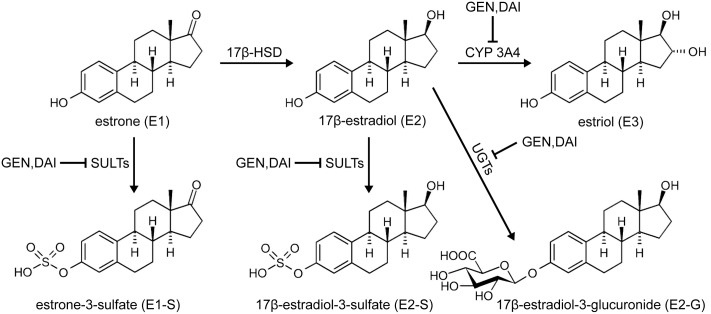
Effect of soy isoflavones on estrogen metabolism in MCF-7 cells. Genistein (GEN) and daidzein (DAI) inhibit the formation of E1-S, E2-S, E2-G and E3, thereby increasing active E2 levels.

Concomitant with the observed inhibition of E1 and E2 conjugation, genistein and daidzein caused a minor, but statistically significant increase of approximately 20% in the active E2 levels (**Figure 5**). Based on increased E2 formation in the presence of genistein and daidzein, inhibition of 17β-HSD can be excluded. Our data are in contrast to a very recent study showing an inhibition of this enzyme by genistein (Cassetta et al., 2017). This discrepancy might be explained by the fact that the authors used purified recombinant 17β-HSD from the filantous fungus *Cochliobolus lunatus* and not a human enzyme which might differ in activity. Both isoflavones, however, significantly inhibited the activity of cellular SULTs responsible for the formation of E1-S thereby increasing the E1 pool and consequently leading to a higher E2-formation by 17β-HSD. Furthermore, genistein and daidzein also demonstrated a pronounced inhibition of E2-S, E2-G and E3 formation thereby contributing to the observed increased E2 level (**Figure 6**).

The observed increase in E2 in our *in vitro* model was also found in a previous human trial which examined the effect of soy foods on urinary estrogens in premenopausal women (Maskarinec et al., 2012). Participants who consumed a high-soy diet for 13 months showed a non-significant increase of urinary E2 levels of 18%. These findings were confirmed by a meta-analysis (Hooper et al., 2009), in which the authors also reported a small, non-significant increase in total estradiol concentrations of 14% in post-menopausal women following soy isoflavone consumption. Although both studies observed a minor, non-significant increase in urinary E2 levels, soy food consumption should be considered safe, as even the daily intake of two dietary servings of soy powder (25 g each) for up to 30 days lead to mean total plasma levels (parent compound, glucuronides and sulfates) of only 11.6 ng/ml (0.042 μM) for genistein and 6.7 ng/ml (0.026 μM) for daidzein (Shike et al., 2014). These concentrations are far below our calculated *K*_i_ values (0.3–1.6 μM for E1 and E2 sulfates, and 6.0–7.3 μM for E2 glucuronide) therefore suggesting no significant effect of dietary soy intake on estrogen metabolism.

On the other hand, daily high-dose supplementation with genistein (600 mg) and daidzein (300 mg) for 84 days has been found to increase the trough plasma levels up to a concentration of 15 μg/ml (55 μM) total isoflavones (Pop et al., 2008). Taking into account that the majority of isoflavones are extensively metabolized *in vivo* (up to 98%), the remaining free genistein and daidzein plasma concentrations would reach approximately 1 μM, which is still high enough to inhibit E1 and E2 sulfation, while leaving E2 glucuronidation unaffected. Whether genistein or daidzein glucuronides and sulfates also exhibit an inhibitory activity toward E1 and E2 conjugation is not known yet. However, any additive inhibitory effects would further increase the plasma concentration of free E2 following isoflavone supplementation.

## Conclusion

The present work identified a non-competitive inhibition of E1 and E2 conjugation by low micromolar concentrations of soy isoflavones in the human breast cancer cell line MCF-7, which leads to a minor but statistically significant increase in unconjugated E2 of approximately 20%. As the content of genistein and daidzein in soy food is relatively low, an increased risk of breast cancer development and progression in women might only be observed after the continuous consumption of high-dose isoflavone supplements. Further long-term human studies monitoring free estrogens and their conjugates are therefore highly warranted to evaluate the efficacy and safety of high-dose genistein and daidzein supplementation, especially in patients diagnosed with ERα+ breast cancer.

## Author Contributions

SP performed all the cell culture experiments, the LC-HRMS analysis and the data analysis, and contributed to the manuscript. AM-S analyzed the data and contributed to the manuscript. MZ, JW, and DD performed the LC-HRMS analysis. BP and KS cultivated the MCF-7 cells and performed inhibition experiments; and WJ planned the experiments, analyzed the data and wrote the final version of the manuscript.

## Conflict of Interest Statement

The authors declare that the research was conducted in the absence of any commercial or financial relationships that could be construed as a potential conflict of interest.

## Abbreviations

CYP: cytochrome P450 enzyme
DMEM: Dulbecco’s modified Eagle’s medium
DMSO: dimethyl sulfoxide
DPBS: Dulbecco’s phosphate-buffered saline
E1: estrone
E1-S: estrone-3-sulfate
E2: 17β-estradiol
E2-G: 17β-estradiol-3-(β-D-glucuronide)
E2-S: 17β-estradiol-3-sulfate
E3: estriol (16α-OH-17β-estradiol)
EIC: extracted ion chromatogram
ERα: estrogen receptor alpha
ERβ: estrogen receptor beta
ESI: electro-spray ionization
17β-HSD: 17β-hydroxysteroid dehydrogenase
K_i_: inhibition constant
K_m_: Michaelis constant
LC-HRMS: liquid chromatography-high resolution mass spectrometry
LLOQ: lower limit of quantification
SD: standard deviation
SPE: solid phase extraction
SULT: sulfotransferase
UGT: UDP-glucuronosyl transferase
V_max_: maximum reaction velocity
