# Supplementary figures and images for: The Impacts of Genistein and Daidzein on Estrogen Conjugations in Human Breast Cancer Cells: A Targeted Metabolomics Approach
Source: Front Pharmacol. 2017 Oct 5;8:699. doi: 10.3389/fphar.2017.00699 (PMC5633874; doi:10.3389/fphar.2017.00699)

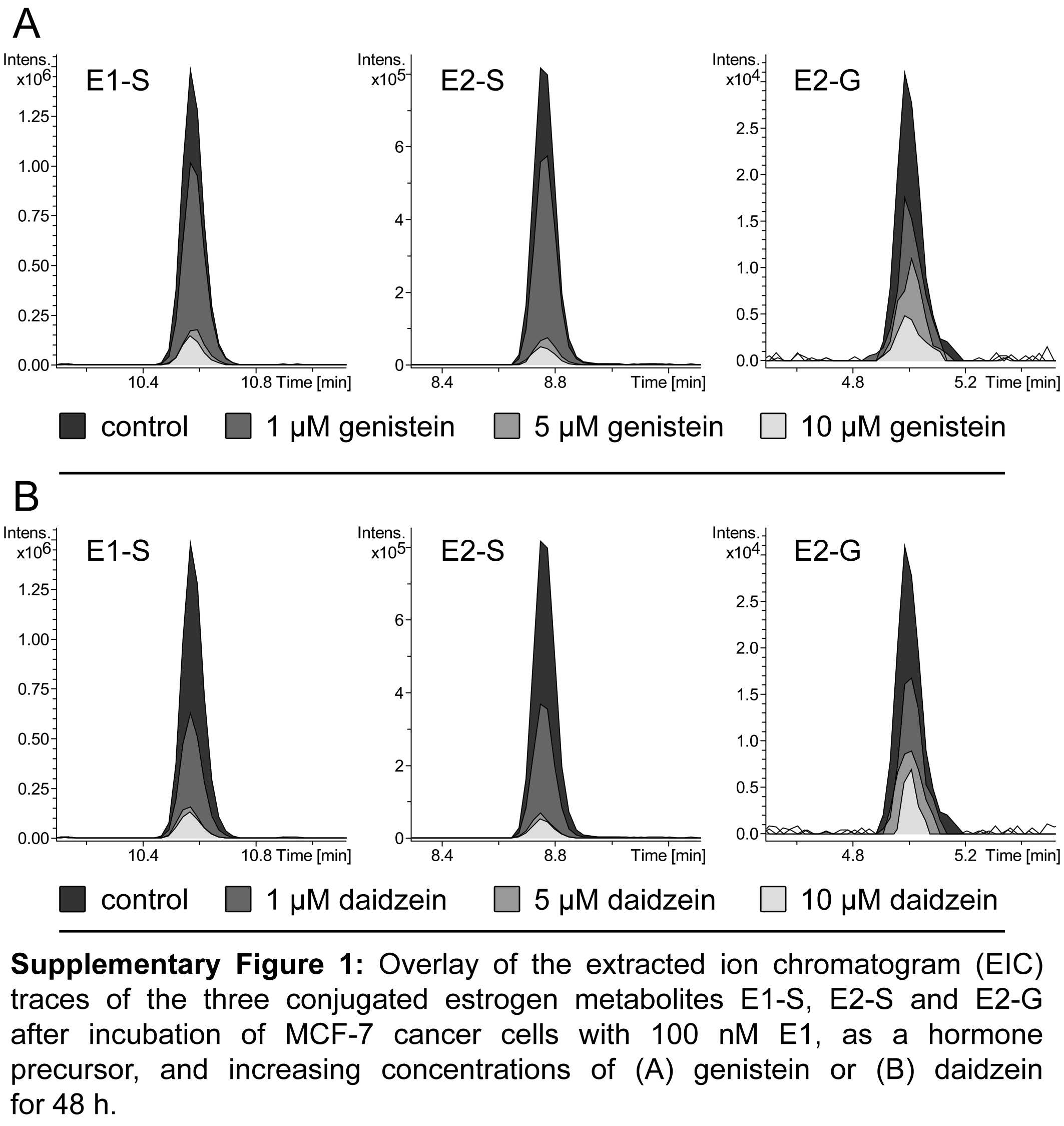

Supplement: Supplementary file 1 [file Image_1.TIF]
